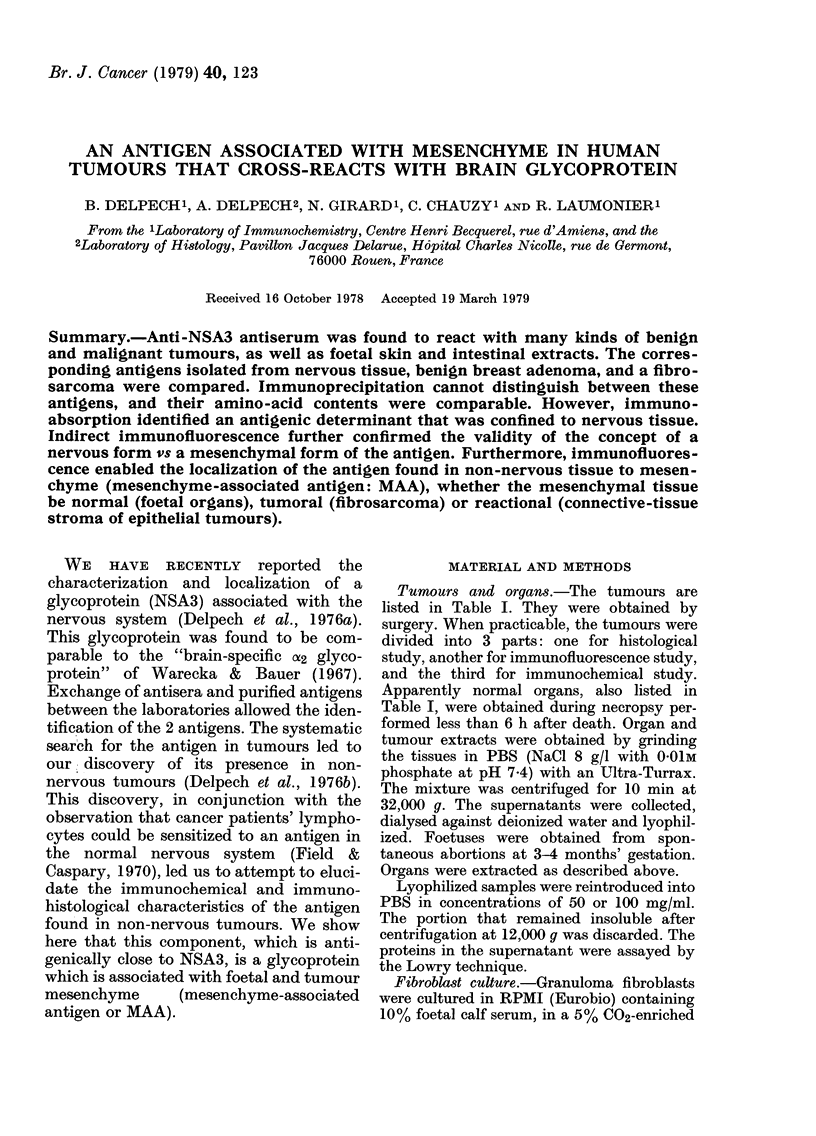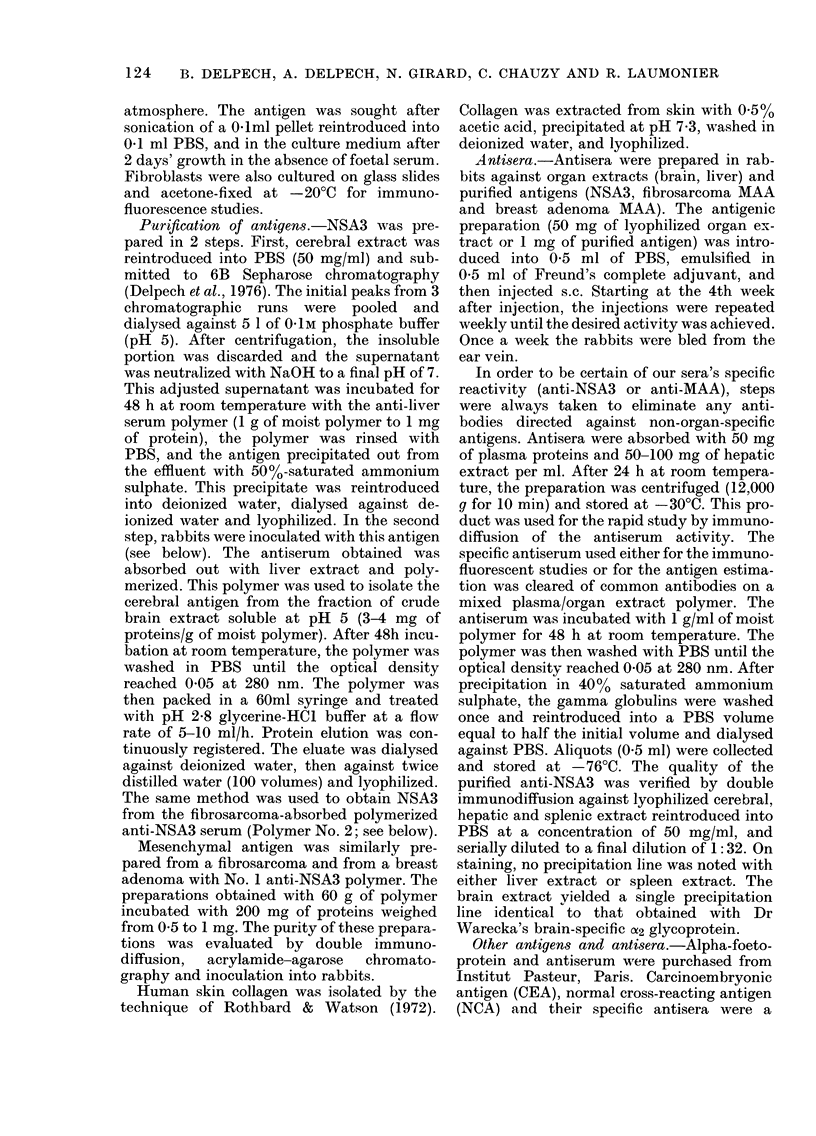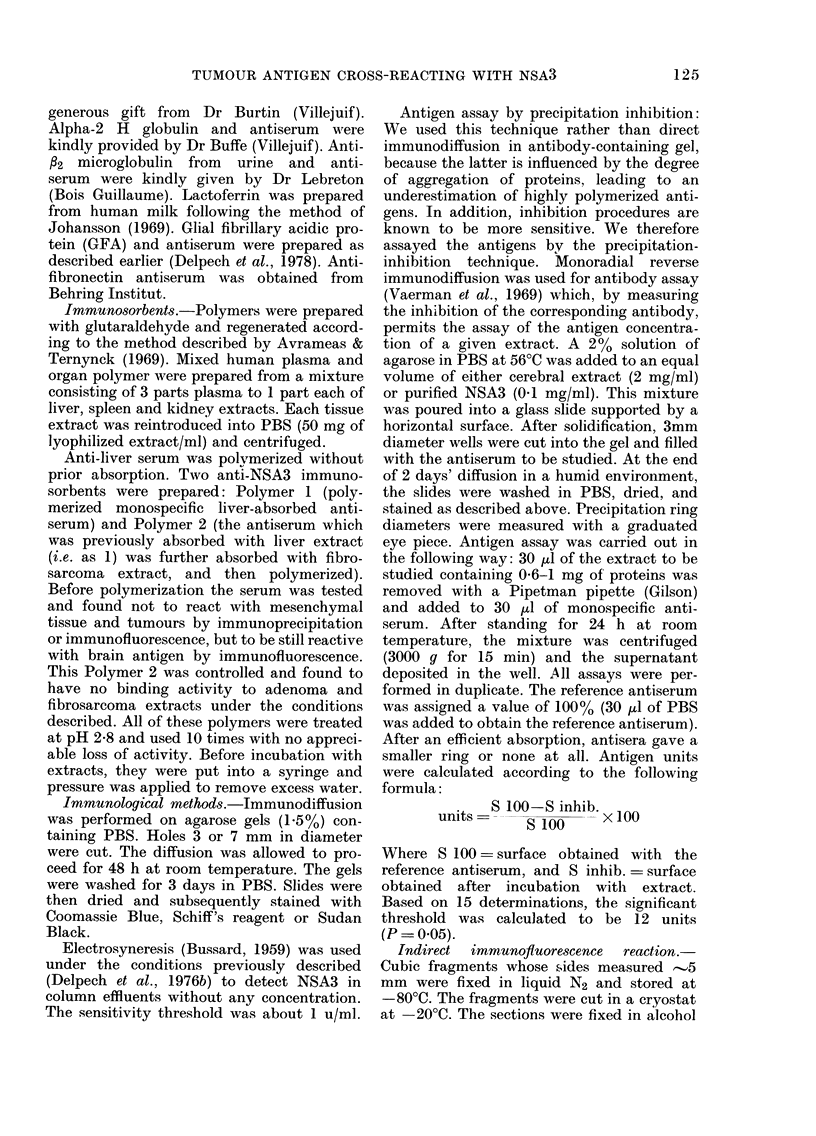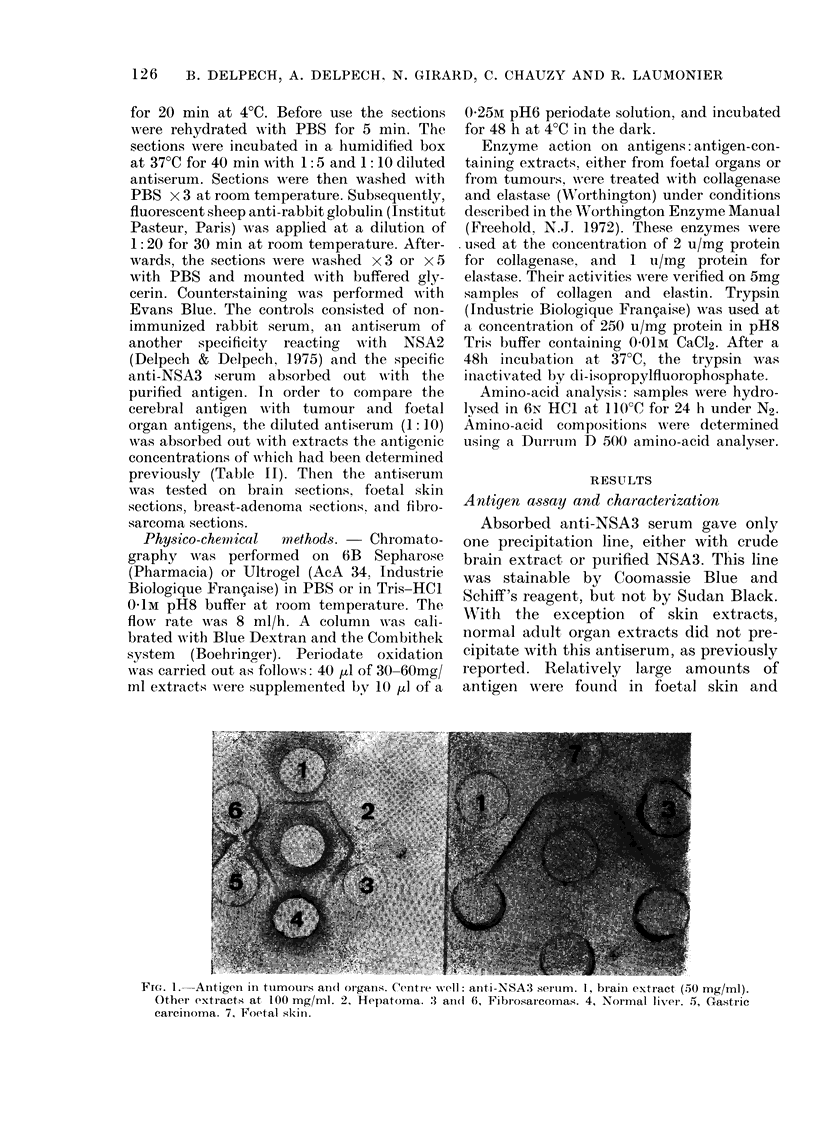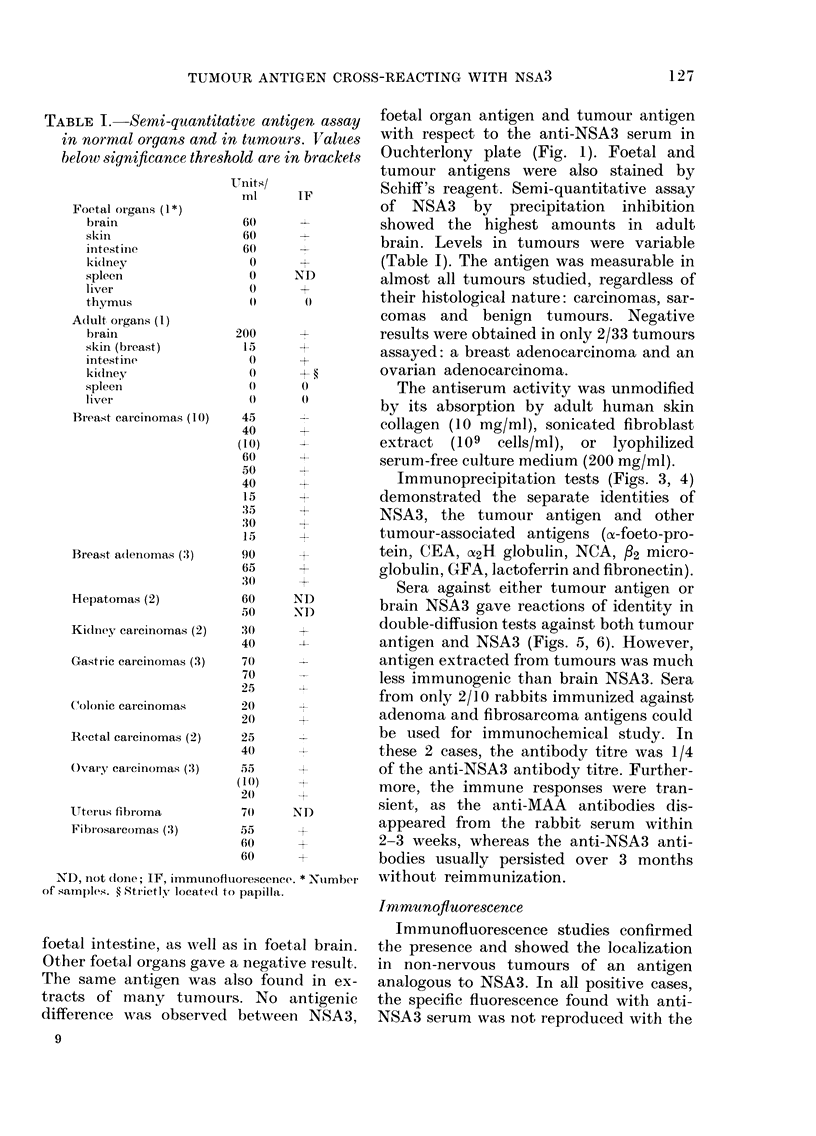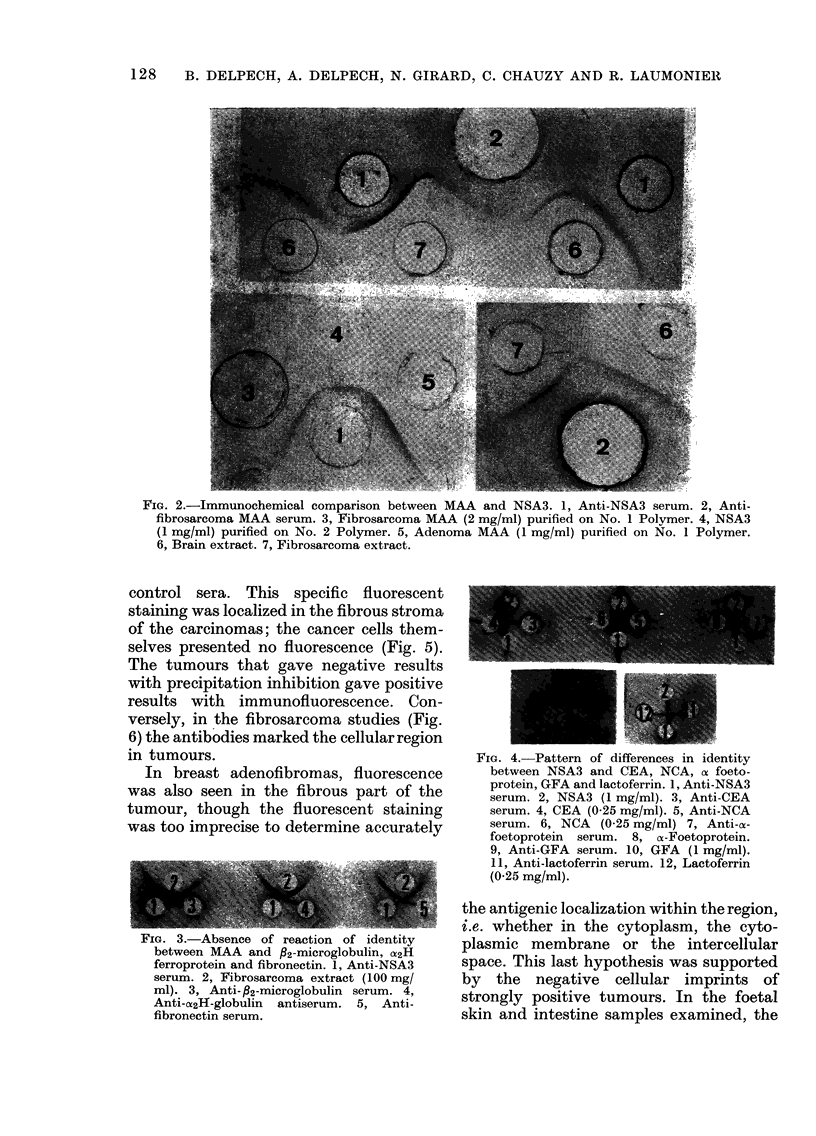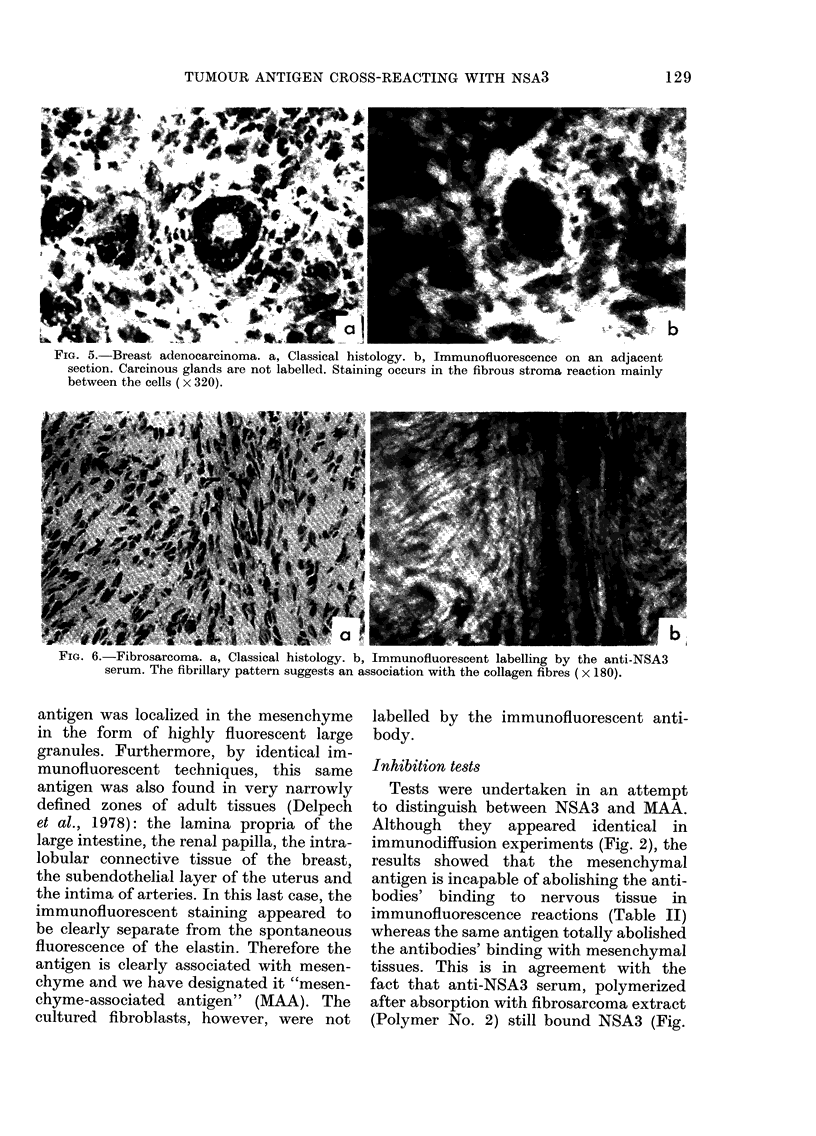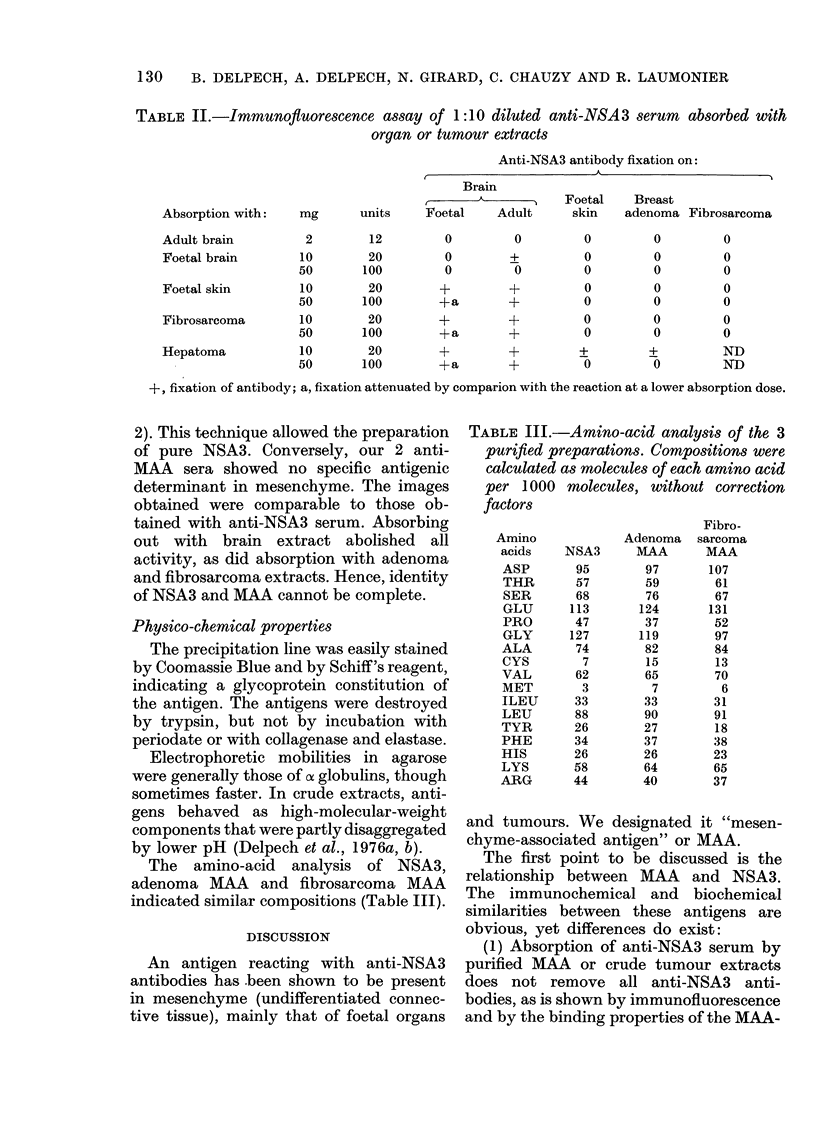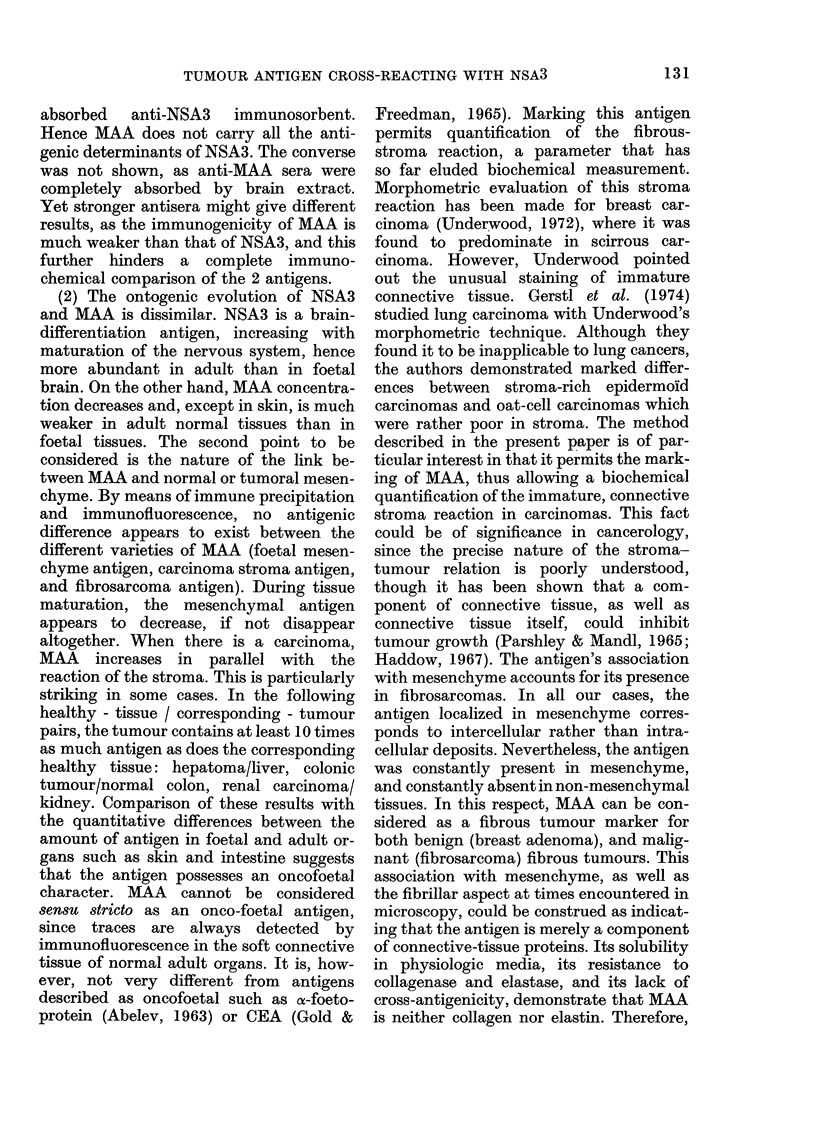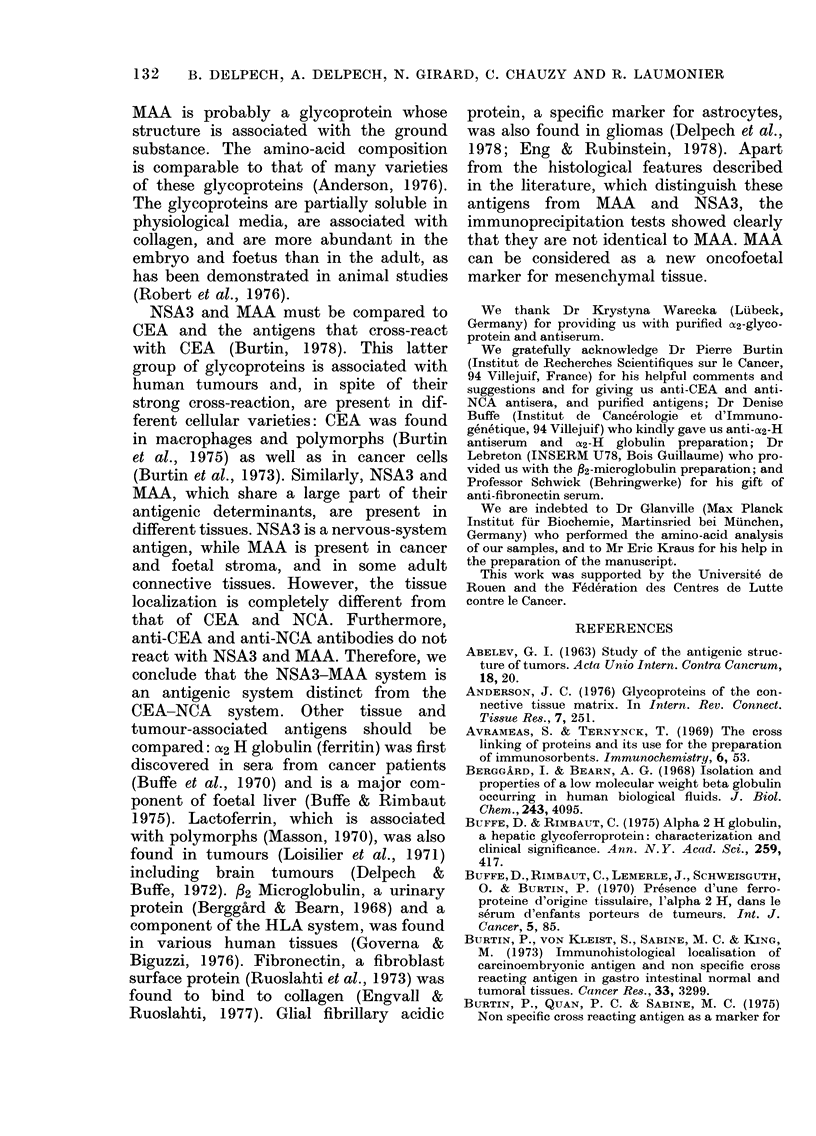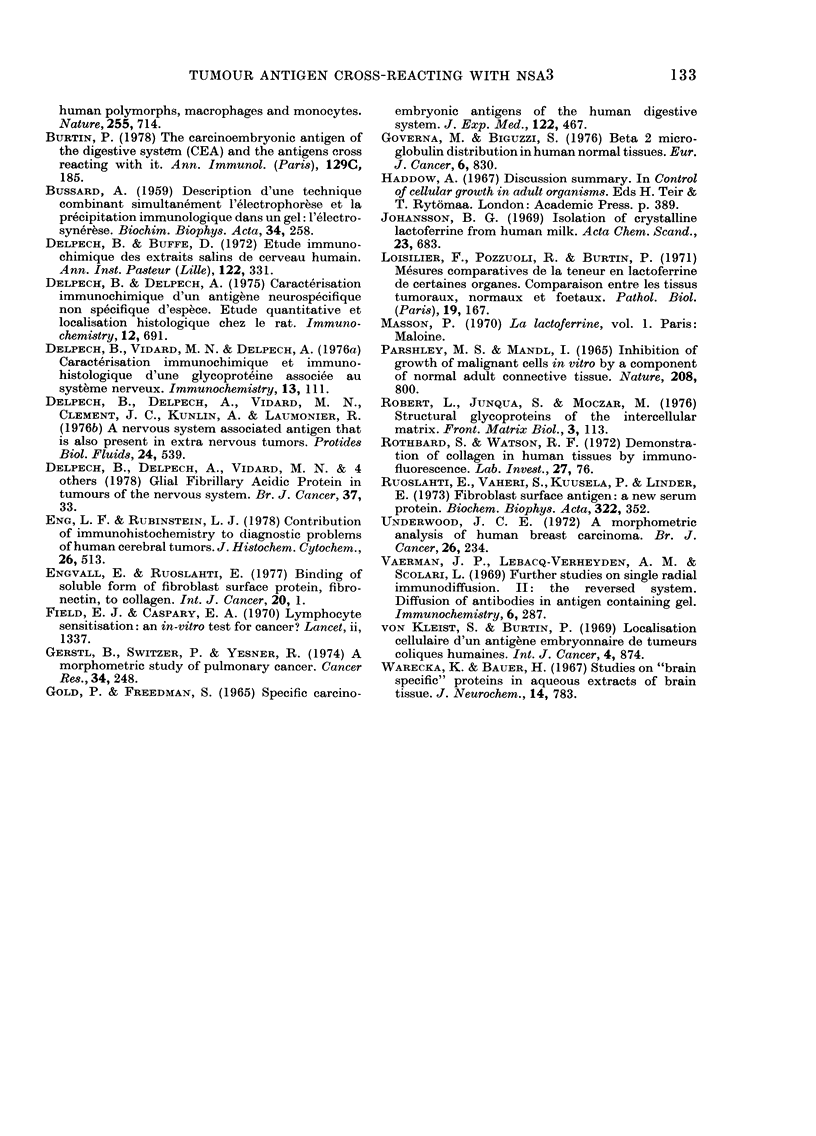# An antigen associated with mesenchyme in human tumours that cross-reacts with brain glycoprotein.

**DOI:** 10.1038/bjc.1979.148

**Published:** 1979-07

**Authors:** B. Delpech, A. Delpech, N. Girard, C. Chauzy, R. Laumonier

## Abstract

**Images:**


*Br. J. Cancer* (1979) **40**, 123

### AN ANTIGEN ASSOCIATED WITH MESENCHYME IN HUMAN TUMOURS THAT CROSS-REACTS WITH BRAIN GLYCOPROTEIN

B. DELPECH[1], A. DELPECH[2], N. GIRARD[1], C. CHAUZY[1] AND R. LAUMONIER[1]

*From the [1]Laboratory of Immunochemistry, Centre Henri Becquerel, rue d'Amiens, and the [2]Laboratory of Histology, Pavillon Jacques Delarue, Hôpital Charles Nicolle, rue de Germont, 76000 Rouen, France*

Received 16 October 1978 Accepted 19 March 1979

**Summary.—Anti-NSA3 antiserum was found to react with many kinds of benign and malignant tumours, as well as foetal skin and intestinal extracts. The corresponding antigens isolated from nervous tissue, benign breast adenoma, and a fibrosarcoma were compared. Immunoprecipitation cannot distinguish between these antigens, and their amino-acid contents were comparable. However, immunoabsorption identified an antigenic determinant that was confined to nervous tissue. Indirect immunofluorescence further confirmed the validity of the concept of a nervous form *vs* a mesenchymal form of the antigen. Furthermore, immunofluorescence enabled the localization of the antigen found in non-nervous tissue to mesenchyme (mesenchyme-associated antigen: MAA), whether the mesenchymal tissue be normal (foetal organs), tumoral (fibrosarcoma) or reactional (connective-tissue stroma of epithelial tumours).**

WE HAVE RECENTLY reported the characterization and localization of a glycoprotein (NSA3) associated with the nervous system (Delpech *et al.*, 1976*a*). This glycoprotein was found to be comparable to the "brain-specific $\alpha_2$ glycoprotein" of Warecka & Bauer (1967). Exchange of antisera and purified antigens between the laboratories allowed the identification of the 2 antigens. The systematic search for the antigen in tumours led to our discovery of its presence in non-nervous tumours (Delpech *et al.*, 1976*b*). This discovery, in conjunction with the observation that cancer patients' lymphocytes could be sensitized to an antigen in the normal nervous system (Field & Caspary, 1970), led us to attempt to elucidate the immunochemical and immunohistological characteristics of the antigen found in non-nervous tumours. We show here that this component, which is antigenically close to NSA3, is a glycoprotein which is associated with foetal and tumour mesenchyme (mesenchyme-associated antigen or MAA).

### MATERIAL AND METHODS

*Tumours and organs.*—The tumours are listed in Table I. They were obtained by surgery. When practicable, the tumours were divided into 3 parts: one for histological study, another for immunofluorescence study, and the third for immunochemical study. Apparently normal organs, also listed in Table I, were obtained during necropsy performed less than 6 h after death. Organ and tumour extracts were obtained by grinding the tissues in PBS (NaCl 8 g/l with 0·01M phosphate at pH 7·4) with an Ultra-Turrax. The mixture was centrifuged for 10 min at 32,000 *g*. The supernatants were collected, dialysed against deionized water and lyophilized. Foetuses were obtained from spontaneous abortions at 3–4 months' gestation. Organs were extracted as described above.

Lyophilized samples were reintroduced into PBS in concentrations of 50 or 100 mg/ml. The portion that remained insoluble after centrifugation at 12,000 *g* was discarded. The proteins in the supernatant were assayed by the Lowry technique.

*Fibroblast culture.*—Granuloma fibroblasts were cultured in RPMI (Eurobio) containing 10% foetal calf serum, in a 5% $CO_2$-enriched atmosphere. The antigen was sought after sonication of a 0·1ml pellet reintroduced into 0·1 ml PBS, and in the culture medium after 2 days' growth in the absence of foetal serum. Fibroblasts were also cultured on glass slides and acetone-fixed at −20°C for immunofluorescence studies.

*Purification of antigens.*—NSA3 was prepared in 2 steps. First, cerebral extract was reintroduced into PBS (50 mg/ml) and submitted to 6B Sepharose chromatography (Delpech *et al.*, 1976). The initial peaks from 3 chromatographic runs were pooled and dialysed against 5 l of 0·1M phosphate buffer (pH 5). After centrifugation, the insoluble portion was discarded and the supernatant was neutralized with NaOH to a final pH of 7. This adjusted supernatant was incubated for 48 h at room temperature with the anti-liver serum polymer (1 g of moist polymer to 1 mg of protein), the polymer was rinsed with PBS, and the antigen precipitated out from the effluent with 50%-saturated ammonium sulphate. This precipitate was reintroduced into deionized water, dialysed against deionized water and lyophilized. In the second step, rabbits were inoculated with this antigen (see below). The antiserum obtained was absorbed out with liver extract and polymerized. This polymer was used to isolate the cerebral antigen from the fraction of crude brain extract soluble at pH 5 (3–4 mg of proteins/g of moist polymer). After 48h incubation at room temperature, the polymer was washed in PBS until the optical density reached 0·05 at 280 nm. The polymer was then packed in a 60ml syringe and treated with pH 2·8 glycerine-HCl buffer at a flow rate of 5–10 ml/h. Protein elution was continuously registered. The eluate was dialysed against deionized water, then against twice distilled water (100 volumes) and lyophilized. The same method was used to obtain NSA3 from the fibrosarcoma-absorbed polymerized anti-NSA3 serum (Polymer No. 2; see below).

Mesenchymal antigen was similarly prepared from a fibrosarcoma and from a breast adenoma with No. 1 anti-NSA3 polymer. The preparations obtained with 60 g of polymer incubated with 200 mg of proteins weighed from 0·5 to 1 mg. The purity of these preparations was evaluated by double immunodiffusion, acrylamide–agarose chromatography and inoculation into rabbits.

Human skin collagen was isolated by the technique of Rothbard & Watson (1972). Collagen was extracted from skin with 0·5% acetic acid, precipitated at pH 7·3, washed in deionized water, and lyophilized.

*Antisera.*—Antisera were prepared in rabbits against organ extracts (brain, liver) and purified antigens (NSA3, fibrosarcoma MAA and breast adenoma MAA). The antigenic preparation (50 mg of lyophilized organ extract or 1 mg of purified antigen) was introduced into 0·5 ml of PBS, emulsified in 0·5 ml of Freund's complete adjuvant, and then injected s.c. Starting at the 4th week after injection, the injections were repeated weekly until the desired activity was achieved. Once a week the rabbits were bled from the ear vein.

In order to be certain of our sera's specific reactivity (anti-NSA3 or anti-MAA), steps were always taken to eliminate any antibodies directed against non-organ-specific antigens. Antisera were absorbed with 50 mg of plasma proteins and 50–100 mg of hepatic extract per ml. After 24 h at room temperature, the preparation was centrifuged (12,000 *g* for 10 min) and stored at −30°C. This product was used for the rapid study by immunodiffusion of the antiserum activity. The specific antiserum used either for the immunofluorescent studies or for the antigen estimation was cleared of common antibodies on a mixed plasma/organ extract polymer. The antiserum was incubated with 1 g/ml of moist polymer for 48 h at room temperature. The polymer was then washed with PBS until the optical density reached 0·05 at 280 nm. After precipitation in 40% saturated ammonium sulphate, the gamma globulins were washed once and reintroduced into a PBS volume equal to half the initial volume and dialysed against PBS. Aliquots (0·5 ml) were collected and stored at −76°C. The quality of the purified anti-NSA3 was verified by double immunodiffusion against lyophilized cerebral, hepatic and splenic extract reintroduced into PBS at a concentration of 50 mg/ml, and serially diluted to a final dilution of 1:32. On staining, no precipitation line was noted with either liver extract or spleen extract. The brain extract yielded a single precipitation line identical to that obtained with Dr Warecka's brain-specific $\alpha_2$ glycoprotein.

*Other antigens and antisera.*—Alpha-foetoprotein and antiserum were purchased from Institut Pasteur, Paris. Carcinoembryonic antigen (CEA), normal cross-reacting antigen (NCA) and their specific antisera were a generous gift from Dr Burtin (Villejuif). Alpha-2 H globulin and antiserum were kindly provided by Dr Buffe (Villejuif). Anti-$\beta_2$ microglobulin from urine and antiserum were kindly given by Dr Lebreton (Bois Guillaume). Lactoferrin was prepared from human milk following the method of Johansson (1969). Glial fibrillary acidic protein (GFA) and antiserum were prepared as described earlier (Delpech *et al.*, 1978). Anti-fibronectin antiserum was obtained from Behring Institut.

*Immunosorbents.*—Polymers were prepared with glutaraldehyde and regenerated according to the method described by Avrameas & Ternynck (1969). Mixed human plasma and organ polymer were prepared from a mixture consisting of 3 parts plasma to 1 part each of liver, spleen and kidney extracts. Each tissue extract was reintroduced into PBS (50 mg of lyophilized extract/ml) and centrifuged.

Anti-liver serum was polymerized without prior absorption. Two anti-NSA3 immunosorbents were prepared: Polymer 1 (polymerized monospecific liver-absorbed antiserum) and Polymer 2 (the antiserum which was previously absorbed with liver extract (*i.e.* as 1) was further absorbed with fibrosarcoma extract, and then polymerized). Before polymerization the serum was tested and found not to react with mesenchymal tissue and tumours by immunoprecipitation or immunofluorescence, but to be still reactive with brain antigen by immunofluorescence. This Polymer 2 was controlled and found to have no binding activity to adenoma and fibrosarcoma extracts under the conditions described. All of these polymers were treated at pH 2·8 and used 10 times with no appreciable loss of activity. Before incubation with extracts, they were put into a syringe and pressure was applied to remove excess water.

*Immunological methods.*—Immunodiffusion was performed on agarose gels (1·5%) containing PBS. Holes 3 or 7 mm in diameter were cut. The diffusion was allowed to proceed for 48 h at room temperature. The gels were washed for 3 days in PBS. Slides were then dried and subsequently stained with Coomassie Blue, Schiff's reagent or Sudan Black.

Electrosyneresis (Bussard, 1959) was used under the conditions previously described (Delpech *et al.*, 1976*b*) to detect NSA3 in column effluents without any concentration. The sensitivity threshold was about 1 u/ml.

Antigen assay by precipitation inhibition: We used this technique rather than direct immunodiffusion in antibody-containing gel, because the latter is influenced by the degree of aggregation of proteins, leading to an underestimation of highly polymerized antigens. In addition, inhibition procedures are known to be more sensitive. We therefore assayed the antigens by the precipitation-inhibition technique. Monoradial reverse immunodiffusion was used for antibody assay (Vaerman *et al.*, 1969) which, by measuring the inhibition of the corresponding antibody, permits the assay of the antigen concentration of a given extract. A 2% solution of agarose in PBS at 56°C was added to an equal volume of either cerebral extract (2 mg/ml) or purified NSA3 (0·1 mg/ml). This mixture was poured into a glass slide supported by a horizontal surface. After solidification, 3mm diameter wells were cut into the gel and filled with the antiserum to be studied. At the end of 2 days' diffusion in a humid environment, the slides were washed in PBS, dried, and stained as described above. Precipitation ring diameters were measured with a graduated eye piece. Antigen assay was carried out in the following way: 30 $\mu$l of the extract to be studied containing 0·6–1 mg of proteins was removed with a Pipetman pipette (Gilson) and added to 30 $\mu$l of monospecific antiserum. After standing for 24 h at room temperature, the mixture was centrifuged (3000 *g* for 15 min) and the supernatant deposited in the well. All assays were performed in duplicate. The reference antiserum was assigned a value of 100% (30 $\mu$l of PBS was added to obtain the reference antiserum). After an efficient absorption, antisera gave a smaller ring or none at all. Antigen units were calculated according to the following formula:

$$\text{units} = \frac{\text{S }100 - \text{S inhib.}}{\text{S }100} \times 100$$

Where S 100 = surface obtained with the reference antiserum, and S inhib. = surface obtained after incubation with extract. Based on 15 determinations, the significant threshold was calculated to be 12 units ($P = 0{\cdot}05$).

*Indirect immunofluorescence reaction.*—Cubic fragments whose sides measured ~5 mm were fixed in liquid $N_2$ and stored at −80°C. The fragments were cut in a cryostat at −20°C. The sections were fixed in alcohol for 20 min at 4°C. Before use the sections were rehydrated with PBS for 5 min. The sections were incubated in a humidified box at 37°C for 40 min with 1:5 and 1:10 diluted antiserum. Sections were then washed with PBS ×3 at room temperature. Subsequently, fluorescent sheep anti-rabbit globulin (Institut Pasteur, Paris) was applied at a dilution of 1:20 for 30 min at room temperature. Afterwards, the sections were washed ×3 or ×5 with PBS and mounted with buffered glycerin. Counterstaining was performed with Evans Blue. The controls consisted of non-immunized rabbit serum, an antiserum of another specificity reacting with NSA2 (Delpech & Delpech, 1975) and the specific anti-NSA3 serum absorbed out with the purified antigen. In order to compare the cerebral antigen with tumour and foetal organ antigens, the diluted antiserum (1:10) was absorbed out with extracts the antigenic concentrations of which had been determined previously (Table II). Then the antiserum was tested on brain sections, foetal skin sections, breast-adenoma sections, and fibrosarcoma sections.

*Physico-chemical methods.* — Chromatography was performed on 6B Sepharose (Pharmacia) or Ultrogel (AcA 34, Industrie Biologique Française) in PBS or in Tris–HCl 0·1M pH8 buffer at room temperature. The flow rate was 8 ml/h. A column was calibrated with Blue Dextran and the Combithek system (Boehringer). Periodate oxidation was carried out as follows: 40 $\mu$l of 30–60mg/ml extracts were supplemented by 10 $\mu$l of a 0·25M pH6 periodate solution, and incubated for 48 h at 4°C in the dark.

Enzyme action on antigens: antigen-containing extracts, either from foetal organs or from tumours, were treated with collagenase and elastase (Worthington) under conditions described in the Worthington Enzyme Manual (Freehold, N.J. 1972). These enzymes were used at the concentration of 2 u/mg protein for collagenase, and 1 u/mg protein for elastase. Their activities were verified on 5mg samples of collagen and elastin. Trypsin (Industrie Biologique Française) was used at a concentration of 250 u/mg protein in pH8 Tris buffer containing 0·01M $CaCl_2$. After a 48h incubation at 37°C, the trypsin was inactivated by di-isopropylfluorophosphate.

Amino-acid analysis: samples were hydrolysed in 6N HCl at 110°C for 24 h under $N_2$. Amino-acid compositions were determined using a Durrum D 500 amino-acid analyser.

### RESULTS

### *Antigen assay and characterization*

Absorbed anti-NSA3 serum gave only one precipitation line, either with crude brain extract or purified NSA3. This line was stainable by Coomassie Blue and Schiff's reagent, but not by Sudan Black. With the exception of skin extracts, normal adult organ extracts did not precipitate with this antiserum, as previously reported. Relatively large amounts of antigen were found in foetal skin and

FIG. 1.—Antigen in tumours and organs. Centre well: anti-NSA3 serum. 1, brain extract (50 mg/ml). Other extracts at 100 mg/ml. 2, Hepatoma. 3 and 6, Fibrosarcomas. 4, Normal liver. 5, Gastric carcinoma. 7, Foetal skin.

TABLE I.—*Semi-quantitative antigen assay in normal organs and in tumours. Values below significance threshold are in brackets*

| | Units/ml | IF |
|---|---|---|
| Foetal organs (1*) | | |
| brain | 60 | + |
| skin | 60 | + |
| intestine | 60 | + |
| kidney | 0 | + |
| spleen | 0 | ND |
| liver | 0 | + |
| thymus | 0 | 0 |
| Adult organs (1) | | |
| brain | 200 | + |
| skin (breast) | 15 | + |
| intestine | 0 | + |
| kidney | 0 | + § |
| spleen | 0 | 0 |
| liver | 0 | 0 |
| Breast carcinomas (10) | 45 | + |
| | 40 | + |
| | (10) | + |
| | 60 | + |
| | 50 | + |
| | 40 | + |
| | 15 | + |
| | 35 | + |
| | 30 | + |
| | 15 | + |
| Breast adenomas (3) | 90 | + |
| | 65 | + |
| | 30 | + |
| Hepatomas (2) | 60 | ND |
| | 50 | ND |
| Kidney carcinomas (2) | 30 | + |
| | 40 | + |
| Gastric carcinomas (3) | 70 | + |
| | 70 | + |
| | 25 | + |
| Colonic carcinomas | 20 | + |
| | 20 | + |
| Rectal carcinomas (2) | 25 | + |
| | 40 | + |
| Ovary carcinomas (3) | 55 | + |
| | (10) | + |
| | 20 | + |
| Uterus fibroma | 70 | ND |
| Fibrosarcomas (3) | 55 | + |
| | 60 | + |
| | 60 | + |

ND, not done; IF, immunofluorescence. * Number of samples. § Strictly located to papilla.

foetal intestine, as well as in foetal brain. Other foetal organs gave a negative result. The same antigen was also found in extracts of many tumours. No antigenic difference was observed between NSA3, foetal organ antigen and tumour antigen with respect to the anti-NSA3 serum in Ouchterlony plate (Fig. 1). Foetal and tumour antigens were also stained by Schiff's reagent. Semi-quantitative assay of NSA3 by precipitation inhibition showed the highest amounts in adult brain. Levels in tumours were variable (Table I). The antigen was measurable in almost all tumours studied, regardless of their histological nature: carcinomas, sarcomas and benign tumours. Negative results were obtained in only 2/33 tumours assayed: a breast adenocarcinoma and an ovarian adenocarcinoma.

The antiserum activity was unmodified by its absorption by adult human skin collagen (10 mg/ml), sonicated fibroblast extract ($10^9$ cells/ml), or lyophilized serum-free culture medium (200 mg/ml).

Immunoprecipitation tests (Figs. 3, 4) demonstrated the separate identities of NSA3, the tumour antigen and other tumour-associated antigens ($\alpha$-foeto-protein, CEA, $\alpha_2$H globulin, NCA, $\beta_2$ microglobulin, GFA, lactoferrin and fibronectin).

Sera against either tumour antigen or brain NSA3 gave reactions of identity in double-diffusion tests against both tumour antigen and NSA3 (Figs. 5, 6). However, antigen extracted from tumours was much less immunogenic than brain NSA3. Sera from only 2/10 rabbits immunized against adenoma and fibrosarcoma antigens could be used for immunochemical study. In these 2 cases, the antibody titre was 1/4 of the anti-NSA3 antibody titre. Furthermore, the immune responses were transient, as the anti-MAA antibodies disappeared from the rabbit serum within 2–3 weeks, whereas the anti-NSA3 antibodies usually persisted over 3 months without reimmunization.

*Immunofluorescence*

Immunofluorescence studies confirmed the presence and showed the localization in non-nervous tumours of an antigen analogous to NSA3. In all positive cases, the specific fluorescence found with anti-NSA3 serum was not reproduced with the

FIG. 2.—Immunochemical comparison between MAA and NSA3. 1, Anti-NSA3 serum. 2, Anti-fibrosarcoma MAA serum. 3, Fibrosarcoma MAA (2 mg/ml) purified on No. 1 Polymer. 4, NSA3 (1 mg/ml) purified on No. 2 Polymer. 5, Adenoma MAA (1 mg/ml) purified on No. 1 Polymer. 6, Brain extract. 7, Fibrosarcoma extract.

control sera. This specific fluorescent staining was localized in the fibrous stroma of the carcinomas; the cancer cells themselves presented no fluorescence (Fig. 5). The tumours that gave negative results with precipitation inhibition gave positive results with immunofluorescence. Conversely, in the fibrosarcoma studies (Fig. 6) the antibodies marked the cellular region in tumours.

In breast adenofibromas, fluorescence was also seen in the fibrous part of the tumour, though the fluorescent staining was too imprecise to determine accurately

FIG. 3.—Absence of reaction of identity between MAA and $\beta_2$-microglobulin, $\alpha_2$H ferroprotein and fibronectin. 1, Anti-NSA3 serum. 2, Fibrosarcoma extract (100 mg/ml). 3, Anti-$\beta_2$-microglobulin serum. 4, Anti-$\alpha_2$H-globulin antiserum. 5, Anti-fibronectin serum.

FIG. 4.—Pattern of differences in identity between NSA3 and CEA, NCA, $\alpha$ foetoprotein, GFA and lactoferrin. 1, Anti-NSA3 serum. 2, NSA3 (1 mg/ml). 3, Anti-CEA serum. 4, CEA (0·25 mg/ml). 5, Anti-NCA serum. 6, NCA (0·25 mg/ml) 7, Anti-$\alpha$-foetoprotein serum. 8, $\alpha$-Foetoprotein. 9, Anti-GFA serum. 10, GFA (1 mg/ml). 11, Anti-lactoferrin serum. 12, Lactoferrin (0·25 mg/ml).

the antigenic localization within the region, *i.e.* whether in the cytoplasm, the cytoplasmic membrane or the intercellular space. This last hypothesis was supported by the negative cellular imprints of strongly positive tumours. In the foetal skin and intestine samples examined, the

FIG. 5.—Breast adenocarcinoma. a, Classical histology. b, Immunofluorescence on an adjacent section. Carcinous glands are not labelled. Staining occurs in the fibrous stroma reaction mainly between the cells ( × 320).

FIG. 6.—Fibrosarcoma. a, Classical histology. b, Immunofluorescent labelling by the anti-NSA3 serum. The fibrillary pattern suggests an association with the collagen fibres ( × 180).

antigen was localized in the mesenchyme in the form of highly fluorescent large granules. Furthermore, by identical immunofluorescent techniques, this same antigen was also found in very narrowly defined zones of adult tissues (Delpech *et al.*, 1978): the lamina propria of the large intestine, the renal papilla, the intralobular connective tissue of the breast, the subendothelial layer of the uterus and the intima of arteries. In this last case, the immunofluorescent staining appeared to be clearly separate from the spontaneous fluorescence of the elastin. Therefore the antigen is clearly associated with mesenchyme and we have designated it "mesenchyme-associated antigen" (MAA). The cultured fibroblasts, however, were not labelled by the immunofluorescent antibody.

*Inhibition tests*

Tests were undertaken in an attempt to distinguish between NSA3 and MAA. Although they appeared identical in immunodiffusion experiments (Fig. 2), the results showed that the mesenchymal antigen is incapable of abolishing the antibodies' binding to nervous tissue in immunofluorescence reactions (Table II) whereas the same antigen totally abolished the antibodies' binding with mesenchymal tissues. This is in agreement with the fact that anti-NSA3 serum, polymerized after absorption with fibrosarcoma extract (Polymer No. 2) still bound NSA3 (Fig.

TABLE II.—*Immunofluorescence assay of* 1:10 *diluted anti-NSA*3 *serum absorbed with organ or tumour extracts*

| | | | Anti-NSA3 antibody fixation on: | | | | |
|---|---|---|---|---|---|---|---|
| | | | Brain | | | | |
| Absorption with: | mg | units | Foetal | Adult | Foetal skin | Breast adenoma | Fibrosarcoma |
| Adult brain | 2 | 12 | 0 | 0 | 0 | 0 | 0 |
| Foetal brain | 10 | 20 | 0 | ± | 0 | 0 | 0 |
| | 50 | 100 | 0 | 0 | 0 | 0 | 0 |
| Foetal skin | 10 | 20 | + | + | 0 | 0 | 0 |
| | 50 | 100 | +a | + | 0 | 0 | 0 |
| Fibrosarcoma | 10 | 20 | + | + | 0 | 0 | 0 |
| | 50 | 100 | +a | + | 0 | 0 | 0 |
| Hepatoma | 10 | 20 | + | + | ± | ± | ND |
| | 50 | 100 | +a | + | 0 | 0 | ND |

+, fixation of antibody; a, fixation attenuated by comparion with the reaction at a lower absorption dose.

2). This technique allowed the preparation of pure NSA3. Conversely, our 2 anti-MAA sera showed no specific antigenic determinant in mesenchyme. The images obtained were comparable to those obtained with anti-NSA3 serum. Absorbing out with brain extract abolished all activity, as did absorption with adenoma and fibrosarcoma extracts. Hence, identity of NSA3 and MAA cannot be complete.

*Physico-chemical properties*

The precipitation line was easily stained by Coomassie Blue and by Schiff's reagent, indicating a glycoprotein constitution of the antigen. The antigens were destroyed by trypsin, but not by incubation with periodate or with collagenase and elastase.

Electrophoretic mobilities in agarose were generally those of $\alpha$ globulins, though sometimes faster. In crude extracts, antigens behaved as high-molecular-weight components that were partly disaggregated by lower pH (Delpech *et al.*, 1976*a*, *b*).

The amino-acid analysis of NSA3, adenoma MAA and fibrosarcoma MAA indicated similar compositions (Table III).

TABLE III.—*Amino-acid analysis of the* 3 *purified preparations. Compositions were calculated as molecules of each amino acid per* 1000 *molecules, without correction factors*

| Amino acids | NSA3 | Adenoma MAA | Fibro-sarcoma MAA |
|---|---|---|---|
| ASP | 95 | 97 | 107 |
| THR | 57 | 59 | 61 |
| SER | 68 | 76 | 67 |
| GLU | 113 | 124 | 131 |
| PRO | 47 | 37 | 52 |
| GLY | 127 | 119 | 97 |
| ALA | 74 | 82 | 84 |
| CYS | 7 | 15 | 13 |
| VAL | 62 | 65 | 70 |
| MET | 3 | 7 | 6 |
| ILEU | 33 | 33 | 31 |
| LEU | 88 | 90 | 91 |
| TYR | 26 | 27 | 18 |
| PHE | 34 | 37 | 38 |
| HIS | 26 | 26 | 23 |
| LYS | 58 | 64 | 65 |
| ARG | 44 | 40 | 37 |

### DISCUSSION

An antigen reacting with anti-NSA3 antibodies has been shown to be present in mesenchyme (undifferentiated connective tissue), mainly that of foetal organs and tumours. We designated it "mesenchyme-associated antigen" or MAA.

The first point to be discussed is the relationship between MAA and NSA3. The immunochemical and biochemical similarities between these antigens are obvious, yet differences do exist:

(1) Absorption of anti-NSA3 serum by purified MAA or crude tumour extracts does not remove all anti-NSA3 antibodies, as is shown by immunofluorescence and by the binding properties of the MAA-

absorbed anti-NSA3 immunosorbent. Hence MAA does not carry all the antigenic determinants of NSA3. The converse was not shown, as anti-MAA sera were completely absorbed by brain extract. Yet stronger antisera might give different results, as the immunogenicity of MAA is much weaker than that of NSA3, and this further hinders a complete immunochemical comparison of the 2 antigens.

(2) The ontogenic evolution of NSA3 and MAA is dissimilar. NSA3 is a brain-differentiation antigen, increasing with maturation of the nervous system, hence more abundant in adult than in foetal brain. On the other hand, MAA concentration decreases and, except in skin, is much weaker in adult normal tissues than in foetal tissues. The second point to be considered is the nature of the link between MAA and normal or tumoral mesenchyme. By means of immune precipitation and immunofluorescence, no antigenic difference appears to exist between the different varieties of MAA (foetal mesenchyme antigen, carcinoma stroma antigen, and fibrosarcoma antigen). During tissue maturation, the mesenchymal antigen appears to decrease, if not disappear altogether. When there is a carcinoma, MAA increases in parallel with the reaction of the stroma. This is particularly striking in some cases. In the following healthy - tissue / corresponding - tumour pairs, the tumour contains at least 10 times as much antigen as does the corresponding healthy tissue: hepatoma/liver, colonic tumour/normal colon, renal carcinoma/kidney. Comparison of these results with the quantitative differences between the amount of antigen in foetal and adult organs such as skin and intestine suggests that the antigen possesses an oncofoetal character. MAA cannot be considered *sensu stricto* as an onco-foetal antigen, since traces are always detected by immunofluorescence in the soft connective tissue of normal adult organs. It is, however, not very different from antigens described as oncofoetal such as α-foetoprotein (Abelev, 1963) or CEA (Gold & Freedman, 1965). Marking this antigen permits quantification of the fibrous-stroma reaction, a parameter that has so far eluded biochemical measurement. Morphometric evaluation of this stroma reaction has been made for breast carcinoma (Underwood, 1972), where it was found to predominate in scirrous carcinoma. However, Underwood pointed out the unusual staining of immature connective tissue. Gerstl *et al.* (1974) studied lung carcinoma with Underwood's morphometric technique. Although they found it to be inapplicable to lung cancers, the authors demonstrated marked differences between stroma-rich epidermoïd carcinomas and oat-cell carcinomas which were rather poor in stroma. The method described in the present paper is of particular interest in that it permits the marking of MAA, thus allowing a biochemical quantification of the immature, connective stroma reaction in carcinomas. This fact could be of significance in cancerology, since the precise nature of the stroma–tumour relation is poorly understood, though it has been shown that a component of connective tissue, as well as connective tissue itself, could inhibit tumour growth (Parshley & Mandl, 1965; Haddow, 1967). The antigen's association with mesenchyme accounts for its presence in fibrosarcomas. In all our cases, the antigen localized in mesenchyme corresponds to intercellular rather than intracellular deposits. Nevertheless, the antigen was constantly present in mesenchyme, and constantly absent in non-mesenchymal tissues. In this respect, MAA can be considered as a fibrous tumour marker for both benign (breast adenoma), and malignant (fibrosarcoma) fibrous tumours. This association with mesenchyme, as well as the fibrillar aspect at times encountered in microscopy, could be construed as indicating that the antigen is merely a component of connective-tissue proteins. Its solubility in physiologic media, its resistance to collagenase and elastase, and its lack of cross-antigenicity, demonstrate that MAA is neither collagen nor elastin. Therefore,

MAA is probably a glycoprotein whose structure is associated with the ground substance. The amino-acid composition is comparable to that of many varieties of these glycoproteins (Anderson, 1976). The glycoproteins are partially soluble in physiological media, are associated with collagen, and are more abundant in the embryo and foetus than in the adult, as has been demonstrated in animal studies (Robert *et al.*, 1976).

NSA3 and MAA must be compared to CEA and the antigens that cross-react with CEA (Burtin, 1978). This latter group of glycoproteins is associated with human tumours and, in spite of their strong cross-reaction, are present in different cellular varieties: CEA was found in macrophages and polymorphs (Burtin *et al.*, 1975) as well as in cancer cells (Burtin *et al.*, 1973). Similarly, NSA3 and MAA, which share a large part of their antigenic determinants, are present in different tissues. NSA3 is a nervous-system antigen, while MAA is present in cancer and foetal stroma, and in some adult connective tissues. However, the tissue localization is completely different from that of CEA and NCA. Furthermore, anti-CEA and anti-NCA antibodies do not react with NSA3 and MAA. Therefore, we conclude that the NSA3–MAA system is an antigenic system distinct from the CEA–NCA system. Other tissue and tumour-associated antigens should be compared: $\alpha_2$ H globulin (ferritin) was first discovered in sera from cancer patients (Buffe *et al.*, 1970) and is a major component of foetal liver (Buffe & Rimbaut 1975). Lactoferrin, which is associated with polymorphs (Masson, 1970), was also found in tumours (Loisilier *et al.*, 1971) including brain tumours (Delpech & Buffe, 1972). $\beta_2$ Microglobulin, a urinary protein (Berggård & Bearn, 1968) and a component of the HLA system, was found in various human tissues (Governa & Biguzzi, 1976). Fibronectin, a fibroblast surface protein (Ruoslahti *et al.*, 1973) was found to bind to collagen (Engvall & Ruoslahti, 1977). Glial fibrillary acidic protein, a specific marker for astrocytes, was also found in gliomas (Delpech *et al.*, 1978; Eng & Rubinstein, 1978). Apart from the histological features described in the literature, which distinguish these antigens from MAA and NSA3, the immunoprecipitation tests showed clearly that they are not identical to MAA. MAA can be considered as a new oncofoetal marker for mesenchymal tissue.

We thank Dr Krystyna Warecka (Lübeck, Germany) for providing us with purified $\alpha_2$-glycoprotein and antiserum.

We gratefully acknowledge Dr Pierre Burtin (Institut de Recherches Scientifiques sur le Cancer, 94 Villejuif, France) for his helpful comments and suggestions and for giving us anti-CEA and anti-NCA antisera, and purified antigens; Dr Denise Buffe (Institut de Cancérologie et d'Immunogénétique, 94 Villejuif) who kindly gave us anti-$\alpha_2$-H antiserum and $\alpha_2$-H globulin preparation; Dr Lebreton (INSERM U78, Bois Guillaume) who provided us with the $\beta_2$-microglobulin preparation; and Professor Schwick (Behringwerke) for his gift of anti-fibronectin serum.

We are indebted to Dr Glanville (Max Planck Institut für Biochemie, Martinsried bei München, Germany) who performed the amino-acid analysis of our samples, and to Mr Eric Kraus for his help in the preparation of the manuscript.

This work was supported by the Université de Rouen and the Fédération des Centres de Lutte contre le Cancer.

### REFERENCES

Abelev, G. I. (1963) Study of the antigenic structure of tumors. *Acta Unio Intern. Contra Cancrum*, **18**, 20.

Anderson, J. C. (1976) Glycoproteins of the connective tissue matrix. In *Intern. Rev. Connect. Tissue Res.*, **7**, 251.

Avrameas, S. & Ternynck, T. (1969) The cross linking of proteins and its use for the preparation of immunosorbents. *Immunochemistry*, **6**, 53.

Berggård, I. & Bearn, A. G. (1968) Isolation and properties of a low molecular weight beta globulin occurring in human biological fluids. *J. Biol. Chem.*, **243**, 4095.

Buffe, D. & Rimbaut, C. (1975) Alpha 2 H globulin, a hepatic glycoferroprotein: characterization and clinical significance. *Ann. N.Y. Acad. Sci.*, **259**, 417.

Buffe, D., Rimbaut, C., Lemerle, J., Schweisguth, O. & Burtin, P. (1970) Présence d'une ferroproteine d'origine tissulaire, l'alpha 2 H, dans le sérum d'enfants porteurs de tumeurs. *Int. J. Cancer*, **5**, 85.

Burtin, P., von Kleist, S., Sabine, M. C. & King, M. (1973) Immunohistological localisation of carcinoembryonic antigen and non specific cross reacting antigen in gastro intestinal normal and tumoral tissues. *Cancer Res.*, **33**, 3299.

Burtin, P., Quan, P. C. & Sabine, M. C. (1975) Non specific cross reacting antigen as a marker for human polymorphs, macrophages and monocytes. *Nature*, **255**, 714.

BURTIN, P. (1978) The carcinoembryonic antigen of the digestive system (CEA) and the antigens cross reacting with it. *Ann. Immunol.* (*Paris*), **129C**, 185.

BUSSARD, A. (1959) Description d'une technique combinant simultanément l'électrophorèse et la précipitation immunologique dans un gel: l'électrosynérèse. *Biochim. Biophys. Acta*, **34**, 258.

DELPECH, B. & BUFFE, D. (1972) Etude immunochimique des extraits salins de cerveau humain. *Ann. Inst. Pasteur* (*Lille*), **122**, 331.

DELPECH, B. & DELPECH, A. (1975) Caractérisation immunochimique d'un antigène neurospécifique non spécifique d'espèce. Etude quantitative et localisation histologique chez le rat. *Immunochemistry*, **12**, 691.

DELPECH, B., VIDARD, M. N. & DELPECH, A. (1976*a*) Caractérisation immunochimique et immunohistologique d'une glycoprotéine associée au système nerveux. *Immunochemistry*, **13**, 111.

DELPECH, B., DELPECH, A., VIDARD, M. N., CLEMENT, J. C., KUNLIN, A. & LAUMONIER, R. (1976*b*) A nervous system associated antigen that is also present in extra nervous tumors. *Protides Biol. Fluids*, **24**, 539.

DELPECH, B., DELPECH, A., VIDARD, M. N. & 4 others (1978) Glial Fibrillary Acidic Protein in tumours of the nervous system. *Br. J. Cancer*, **37**, 33.

ENG, L. F. & RUBINSTEIN, L. J. (1978) Contribution of immunohistochemistry to diagnostic problems of human cerebral tumors. *J. Histochem. Cytochem.*, **26**, 513.

ENGVALL, E. & RUOSLAHTI, E. (1977) Binding of soluble form of fibroblast surface protein, fibronectin, to collagen. *Int. J. Cancer*, **20**, 1.

FIELD, E. J. & CASPARY, E. A. (1970) Lymphocyte sensitisation: an *in-vitro* test for cancer? *Lancet*, ii, 1337.

GERSTL, B., SWITZER, P. & YESNER, R. (1974) A morphometric study of pulmonary cancer. *Cancer Res.*, **34**, 248.

GOLD, P. & FREEDMAN, S. (1965) Specific carcinoembryonic antigens of the human digestive system. *J. Exp. Med.*, **122**, 467.

GOVERNA, M. & BIGUZZI, S. (1976) Beta 2 microglobulin distribution in human normal tissues. *Eur. J. Cancer*, **6**, 830.

HADDOW, A. (1967) Discussion summary. In *Control of cellular growth in adult organisms*. Eds H. Teir & T. Rytömaa. London: Academic Press. p. 389.

JOHANSSON, B. G. (1969) Isolation of crystalline lactoferrine from human milk. *Acta Chem. Scand.*, **23**, 683.

LOISILIER, F., POZZUOLI, R. & BURTIN, P. (1971) Mésures comparatives de la teneur en lactoferrine de certaines organes. Comparaison entre les tissus tumoraux, normaux et foetaux. *Pathol. Biol.* (*Paris*), **19**, 167.

MASSON, P. (1970) *La lactoferrine*, vol. 1. Paris: Maloine.

PARSHLEY, M. S. & MANDL, I. (1965) Inhibition of growth of malignant cells *in vitro* by a component of normal adult connective tissue. *Nature*, **208**, 800.

ROBERT, L., JUNQUA, S. & MOCZAR, M. (1976) Structural glycoproteins of the intercellular matrix. *Front. Matrix Biol.*, **3**, 113.

ROTHBARD, S. & WATSON, R. F. (1972) Demonstration of collagen in human tissues by immunofluorescence. *Lab. Invest.*, **27**, 76.

RUOSLAHTI, E., VAHERI, S., KUUSELA, P. & LINDER, E. (1973) Fibroblast surface antigen: a new serum protein. *Biochem. Biophys. Acta*, **322**, 352.

UNDERWOOD, J. C. E. (1972) A morphometric analysis of human breast carcinoma. *Br. J. Cancer*, **26**, 234.

VAERMAN, J. P., LEBACQ-VERHEYDEN, A. M. & SCOLARI, L. (1969) Further studies on single radial immunodiffusion. II: the reversed system. Diffusion of antibodies in antigen containing gel. *Immunochemistry*, **6**, 287.

VON KLEIST, S. & BURTIN, P. (1969) Localisation cellulaire d'un antigène embryonnaire de tumeurs coliques humaines. *Int. J. Cancer*, **4**, 874.

WARECKA, K. & BAUER, H. (1967) Studies on "brain specific" proteins in aqueous extracts of brain tissue. *J. Neurochem.*, **14**, 783.